# Supplementary material for: Inhibitory Effect of a Tankyrase Inhibitor on Mechanical Stress-Induced Protease Expression in Human Articular Chondrocytes
Source: Int J Mol Sci. 2024 Jan 24;25(3):1443. doi: 10.3390/ijms25031443 (PMC10855100; doi:10.3390/ijms25031443)
Supplement: Supplementary file 1 [file ijms-25-01443-s001.zip › ijms-2795958-supplementary.pdf]

Supplementary Figure S1

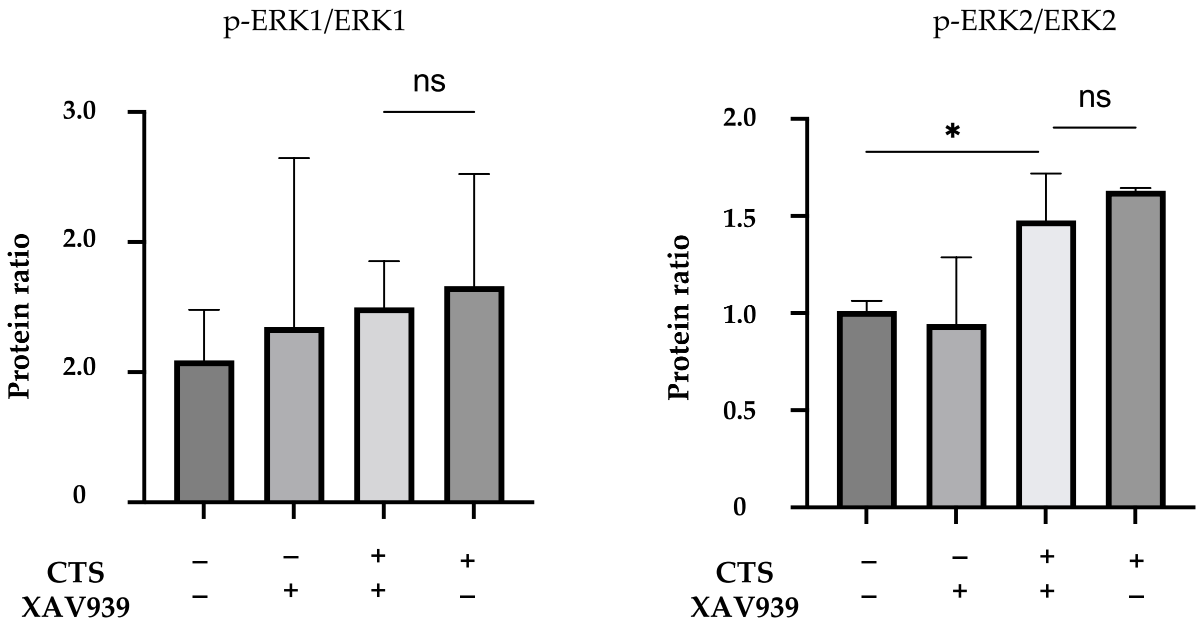

Supplementary Figure S1.

Quantification of the percentage of ERK-1/2 phosphorylation (*$P < 0.05$, ns; not significant). The phosphorylation of ERK2 was significant but ERK1 was not in CTS group compared with control group.

Supplementary Figure S2

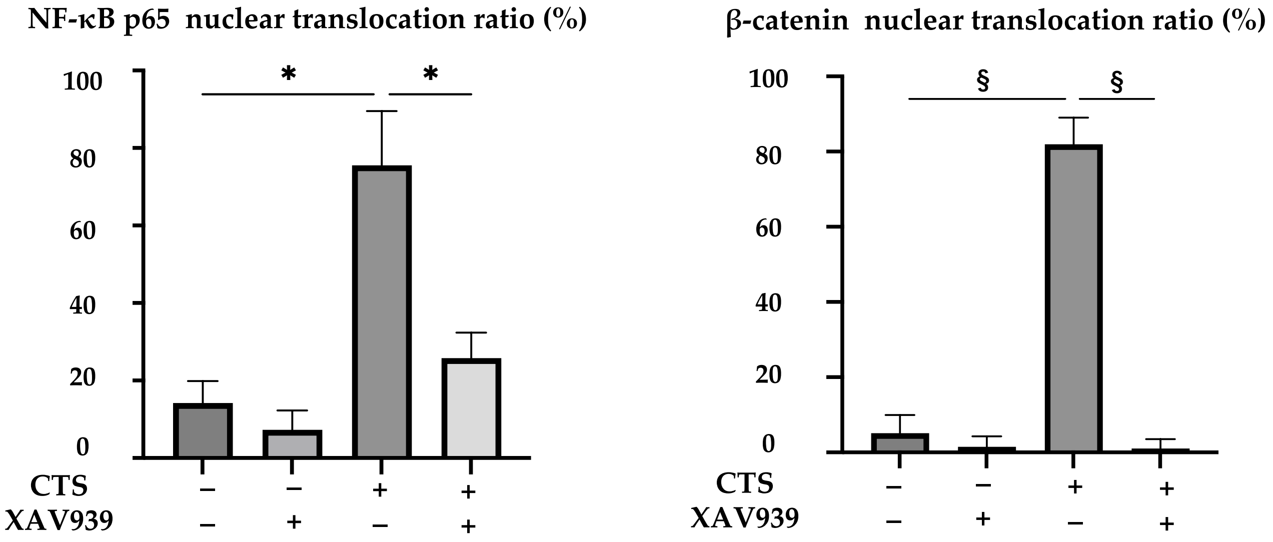

Supplementary Figure S2.

The percentages of chondrocytes positive for nuclear translocation of NF-κB p65 and β-catenin were significantly decreased by treatment with XAV939 by immunocytochemistry (* $P < 0.05$, § $P < 0.0001$).

Supplementary Figure S3

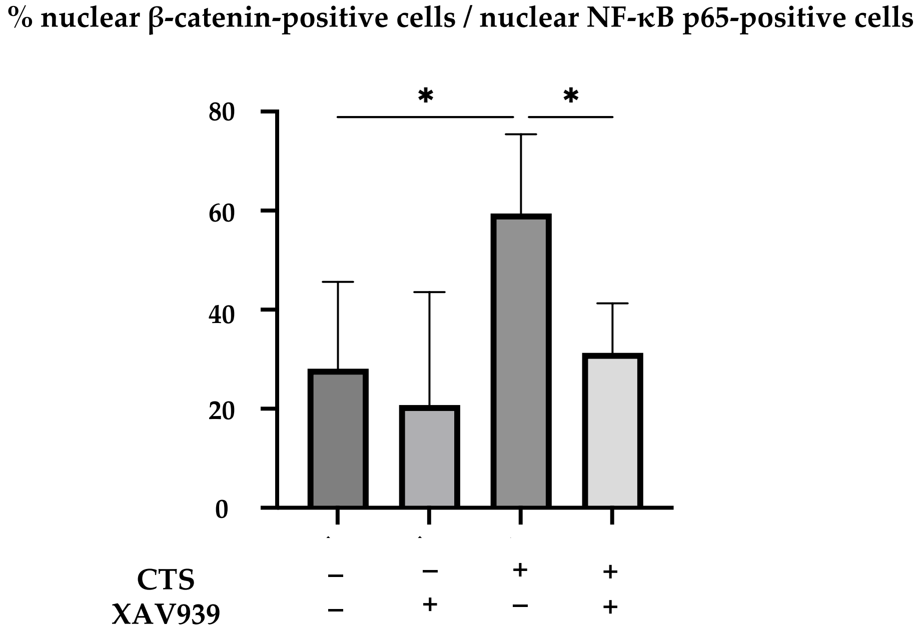

Supplementary Figure S3.

Immunocytochemistry quantification in the form of nuclear β-catenin-positive cells / nuclear NF-κB p65-positive cells was performed (*$P < 0.05$).

Supplementary Figure S4

(A)

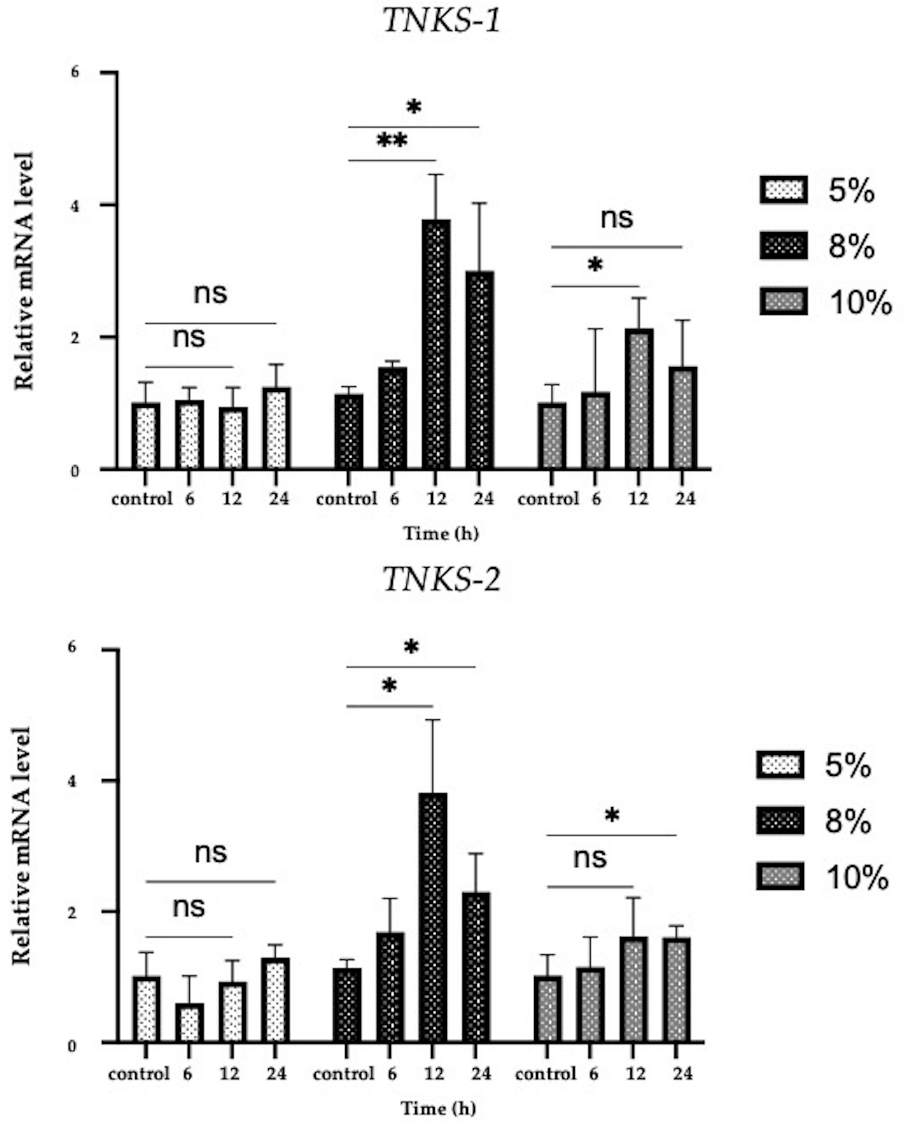

(B)

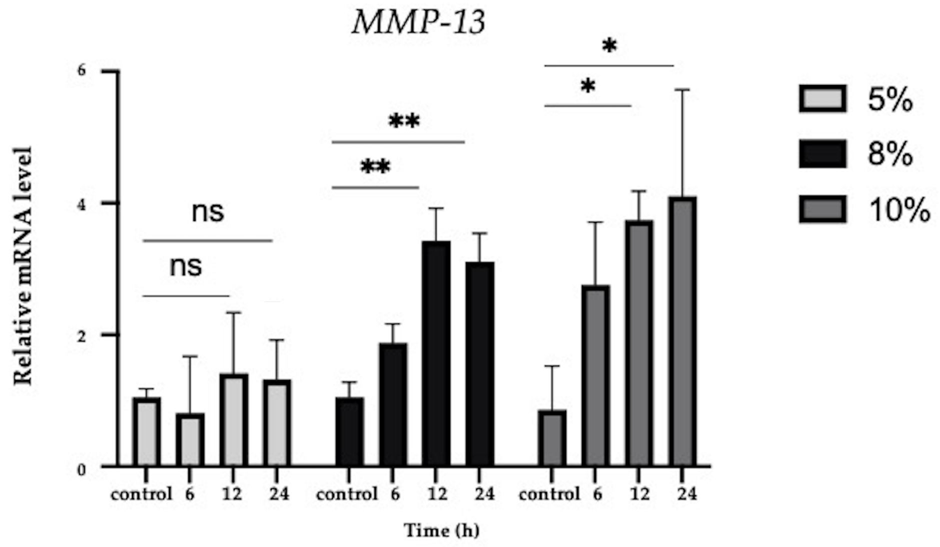

Supplementary Figure S4.

(A) To investigate the appropriate elongation ratio of CTS, we examined the expression of *TNKS-1/2* at different elongation by real-time PCR. The expression of *TNKS-1*/*-2* were significantly increased at 12 h after CTS in 8% elongation compared with control group (*$P < 0.05$, ** $P < 0.01$, ns; not significant).
(B) The expression of *MMP-13* at different elongation. This result means catabolic reaction was promoted at 8 and 10% elongation (*$P < 0.05$, ** $P < 0.01$, ns; not significant).

Supplementary Figure S5

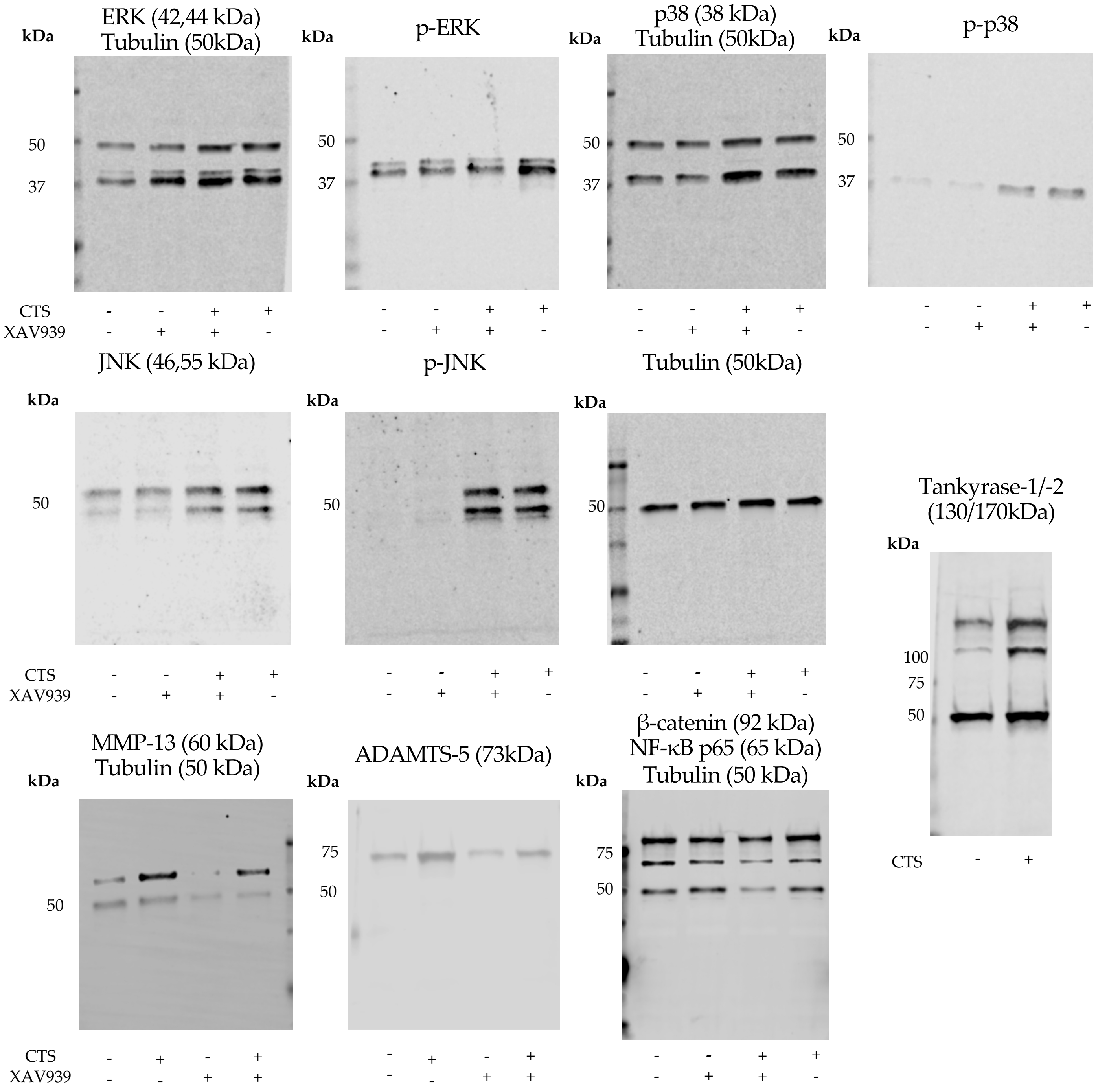

Supplementary Figure S5.

Original, uncropped western blot images for FIgure3. The bands of ERK, p-ERK, p38, p-p38, JNK, p-JNK,, MMP-13, ADAMTS-5, Tankyrase-1/-2, β-catenin, NF-κB p65 and tubulin, and molecular weights of markers in normal human chondrocytes.

Supplementary Table S1

Individual patient data and Mankin's score and Tankyrases expression ratio in articular cartilage.

Case No.	Sample No.	Age	Gender	BMI (kg/m²)	Kellgren Lawrence grade	Mankin's score	Grade	Tankyrases expression ratio (%)		
								Superficial	Middle	Deep
OA 01	01	65	F	33.7	IV	11	Severe	1.1	5.4	3.2
	02					7	Moderate	5.8	34.2	24.4
OA 02	03	64	M	23.6	IV	8	Moderate	22	35.5	38.8
	04					11	Severe	4.5	10.7	9.5
OA 03	05	69	F	16.1	III	9	Moderate	11.1	17.8	35.7
	06					11	Severe	0	16.1	5.7
OA 04	07	61	F	23.5	III	2	Low	0.56	1	0
	08					3	Mild	28.1	5.4	3.7
OA 05	09	73	F	25.5	IV	4	Mild	70	39.5	14.5
OA 06	10	52	F	25.1	III	3	Mild	41.6	9.9	0
OA 07	11	80	F	20.1	III	5	Mild	35	17.6	6.7
OA 08	12	67	F	23.2	III	7	Moderate	8.4	25.9	27.3
OA 09	13	72	M	26.2	IV	8	Moderate	6.6	16.2	36.3
OA 10	14	73	F	29.8	IV	6	Mild	48.9	30	16
OA 11	15	74	M	27.6	IV	1	Low	0	0.4	2
OA 12	16	65	F	25.1	IV	2	Low	1.1	4.1	0.5
OA 13	17	85	F	20.1	III	11	Severe	9.5	11.3	12.5
OA 14	18	67	F	23.2	IV	12	Severe	9.8	19	14.9

Supplementary Table S1.
Tankyrases expression ratio was determined by dividing the number of chondrocytes positive for TNKS-1/2 by the total number of chondrocytes in superficial, middle, and deep layers. The ratio was the average of three fields of view.
BMI ;Body Mass Index
